# Preclinical Efficacy And Safety Evaluation of AAV‐*OTOF* in DFNB9 Mouse Model And Nonhuman Primate

**DOI:** 10.1002/advs.202306201

**Published:** 2023-11-28

**Authors:** Jieyu Qi, Liyan Zhang, Fangzhi Tan, Yang Zhang, Yinyi Zhou, Ziyu Zhang, Hongyang Wang, Chaorong Yu, Lulu Jiang, Jiancheng Liu, Tian Chen, Lianqiu Wu, Shanzhong Zhang, Sijie Sun, Shan Sun, Ling Lu, Qiuju Wang, Renjie Chai

**Affiliations:** ^1^ State Key Laboratory of Digital Medical Engineering Department of Otolaryngology Head and Neck Surgery Zhongda Hospital School of Life Sciences and Technology School of Medicine Advanced Institute for Life and Health Jiangsu Province High‐Tech Key Laboratory for Bio‐Medical Research Southeast University Nanjing 210096 China; ^2^ Co‐Innovation Center of Neuroregeneration Nantong University Nantong 226001 China; ^3^ School of Life Science Beijing Institute of Technology Beijing 100081 China; ^4^ Department of Neurology Affiliated Drum Tower Hospital of Nanjing University Medical School Nanjing 210008 China; ^5^ Senior Department of Otolaryngology‐Head & Neck Surgery the Sixth Medical Center of Chinese PLA General Hospital Beijing 100048 China; ^6^ Otovia Therapeutics Inc. Suzhou 215101 China; ^7^ Fosun Health Capital Shanghai 200233 China; ^8^ ENT Institute and Otorhinolaryngology Department of Affiliated Eye and ENT Hospital Key Laboratory of Hearing Medicine of NHFPC Fudan University Shanghai 200031 China; ^9^ Department of Otolaryngology‐Head and Neck Surgery Jiangsu Provincial Key Medical Discipline (Laboratory) Affiliated Drum Tower Hospital of Nanjing University Medical School Nanjing 210008 China; ^10^ Department of Otolaryngology‐Head and Neck Surgery Sichuan Provincial People's Hospital University of Electronic Science and Technology of China Chengdu 610054 China

**Keywords:** gene therapy, nonhuman primate, OTOF, preclinical research

## Abstract

*OTOF* mutations are the principal causes of auditory neuropathy. There are reports on *Otof*‐related gene therapy in mice, but there is no preclinical research on the drug evaluations. Here, Anc80L65 and the mouse hair cell‐specific Myo15 promoter (mMyo15) are used to selectively and effectively deliver human *OTOF* to hair cells in mice and nonhuman primates to evaluate the efficacy and safety of *OTOF* gene therapy drugs. A new dual‐AAV*‐OTOF‐*hybrid strategy to transfer full‐length *OTOF* is generated, which can stably restore hearing in adult OTOF^p.Q939*/Q939*^ mice with profound deafness, with the longest duration being at least 150 days, and the best therapeutic effect without difference in hearing from wild‐type mice. An AAV microinjection method into the cochlea of cynomolgus monkeys without hearing impairment is further established and found the *OTOF* can be safely and effectively driven by the mMyo15 promoter in hair cells. In addition, the therapeutic dose of AAV drugs has no impact on normal hearing and does not cause significant systemic toxicity both in mouse and nonhuman primates. In summary, this study develops a potential gene therapy strategy for DFNB9 patients in the clinic and provides complete, standardized, and systematic research data for clinical research and application.

## Graphical abstract

This study proposes a gene therapy drug AAV‐*OTOF* and provides preclinical and systematic efficacy and safety evaluation for AAV‐*OTOF* gene therapy both in mice and nonhuman primate, and also establishes a hearing‐safe approach for AAV delivery into the inner ear in nonhuman primate, which can be used to guide the clinical studies of OTOF‐related deafness DFNB9 and provide methodological support for inner ear gene therapies.

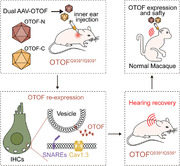

## 1 Introduction

Deafness is a common hereditary disease and can be divided into syndromic hearing loss (SHL) and nonsyndromic hearing loss (NSHL). The incidence of hereditary deafness is very high, with 1–2 out of every 1000 newborns being diagnosed.^[^ ^1^ ^]^ Genetic mutations play a dominant role in the pathogenesis of NSHL, and currently ≈100 genes have been identified as being associated with SHL and NSHL, with an increasing number of causative genes identified every year.^[^ ^2^ ^]^ Mutations in the *OTOF* gene were first identified as the cause of DFNB9, a kind of nonsyndromic recessive auditory neuropathy associated with congenital deafness.^[^ ^3^ ^]^ The *OTOF* gene encodes the OTOF protein, which is highly expressed in inner hair cells (IHCs).^[^ ^4^ ^]^ OTOF is closely associated with synaptic transmission and is thought to be a calcium sensor for exocytosis in IHCs.^[^ ^4^ ^]^ Currently, more than 100 mutant loci in the *OTOF* gene have been reported to cause nonsyndromic deafness DFNB9.^[^ ^5^ ^]^

Currently, most of the treatments for deafness caused by *OTOF* mutations use physical methods such as cochlear implants, and there are no clinically available drugs. Thus, it is urgent to develop biological treatments for hereditary deafness DFNB9. AAV‐based drugs such as Luxturna and Zolgensma have been approved for commercialization by the FDA and are the most common forms of gene therapy in the clinic for patients with single‐gene mutations. Several studies have been conducted on the use of AAV for gene therapy of hereditary deafness, and some specific forms of hereditary hearing loss can be restored by gene delivery or gene editing in hair cells, e.g., deafness caused by *Tmc1* deletion, Ush1c c.216G>A, *Kcnq1* deletion, *Strc* deletion, *Syne4* deletion, Tmc1 c.1253T>A, etc.,^[^ ^6^, ^7^, ^8^, ^9^, ^10^, ^11^, ^12^, ^13^ ^]^ thus indicating the promising application of AAV‐based gene therapy in the treatment of hereditary hearing loss.

The length of the coding region of the *OTOF* gene is 6 kb, which exceeds the packaging limit of AAV (≈4.7 kb).^[^ ^14^ ^]^ Researchers have divided the encoding nucleotide sequence of the *OTOF* gene into two different recombinant AAV vectors, and the full‐length OTOF can be generated by using DNA recombination or protein trans‐splicing in the hair cells of adult mice, and this can restore hearing in *Otof* knockout mice.^[^ ^15^, ^16^, ^17^ ^]^ However, the therapeutic efficacy varies widely in different studies, and there are no studies on the long‐term therapeutic efficacy or safety of *OTOF*‐related gene therapy. The development of informative drug‐forming strategies for AAV‐*OTOF* is the theoretical basis for achieving clinical translation.

The AAV vector consists of two parts, the capsid protein and the gene cassette. The capsid determines the transduction properties of AAV, and the gene cassette determines the expression capability of the transgene. AAV has multiple serotypes, and many have been used for gene delivery in the inner ear. For example, AAV1, AAV8, Anc80L65, and others can transduce almost 100% of adult mouse IHCs.^[^ ^18^, ^19^, ^20^ ^]^ In nonhuman primates (NHPs), PHP.B and Anc80L65 have been reported to efficiently transduce IHCs and are the preferred serotypes for developing *OTOF*‐related gene therapy products.^[^ ^21^, ^22^, ^23^ ^]^ The therapeutic gene cassette usually contains a promoter, the coding sequence, the polyA tail, and other auxiliary enhancer elements such as WPRE. Studies have shown that different promoters have distinct abilities to drive exogenous transgene expression. Some ubiquitous promoters, such as CMV,^[^ ^6^, ^24^ ^]^ CBA (a hybrid cytomegalovirus immediate early/chicken *β*‐actin promoter),^[^ ^15^ ^]^ hbA+CMVe (a human *β*‐actin promoter (hbA)+a cytomegalovirus enhancer), etc.,^[^ ^16^ ^]^ can effectively drive exogenous gene expression in mouse hair cells to achieve therapeutic effects against hearing loss, and CBA and CB7 (an optimized chicken *β*‐actin promoter with an early CMV enhancer) have been shown to drive transgene expression in NHP IHCs.^[^ ^21^, ^22^, ^23^ ^]^ However, in contrast to CMV or specific promoters, another ubiquitous promoter CAG, which has a sequence similar to CBA, is strongly ototoxic in the adult mouse cochlea.^[^ ^25^ ^]^ In contrast, except for hair cells, ubiquitous promoters also drive transgene expression in supporting cells, spiral ganglion neurons, vascular stria cells, etc.^[^ ^18^, ^19^, ^20^, ^26^ ^]^ In addition, AAV usually accumulates in other important organs such as the liver and brain in mice or NHPs.^[^ ^20^, ^21^ ^]^ Due to the immunogenicity and affinity of AAV to nontarget tissues, we speculated that AAV‐OTOF may be potentially toxic to the immune system and organ tissues. Although there is no data showing any cytotoxicity for ubiquitous promoters in NHPs or the human cochlea, choosing an appropriate promoter is one of the key factors for designing safe AAV‐based gene therapy vectors. Using a hair cell‐specific promoter to drive OTOF re‐expression is thus a reasonable strategy because it may reduce AAV expression in other tissues and thus reduce systemic toxicity. However, the published *OTOF* gene therapy studies have all used ubiquitous promoters, and the safety of these vectors has not been reported.

In this study, we used a dual AAV vector strategy to deliver the human OTOF coding sequence into cochlear IHCs with *OTOF* mutations, thus compensating for the loss of OTOF protein and restoring hearing in homozygous OTOF^p.Q939*/Q939*^ mice. We performed preclinical efficacy studies using the OTOF^p.Q939*/Q939*^ mouse model and scientific grade AAV viruses to study *OTOF* splicing sites, AAV serotypes, and promoters in order to optimize the vector for *OTOF* gene therapy. This dual AAV strategy can stably and persistently express full‐length OTOF and restore hearing to wild‐type levels in adult OTOF^p.Q939*/Q939*^ mice with profound deafness. Safety studies in mice and cynomolgus monkeys showed that this dual AAV strategy does not cause significant systemic toxicity or have any short‐term or long‐term effects on auditory, motor, or memory functions. This study provides complete preclinical data for the clinical use of AAV‐mediated gene therapy to restore hearing loss caused by DFNB9.

## 2 Results

### 2.1 Screening of the OTOF Split Site and AAV Serotypes

**Figure 1:**
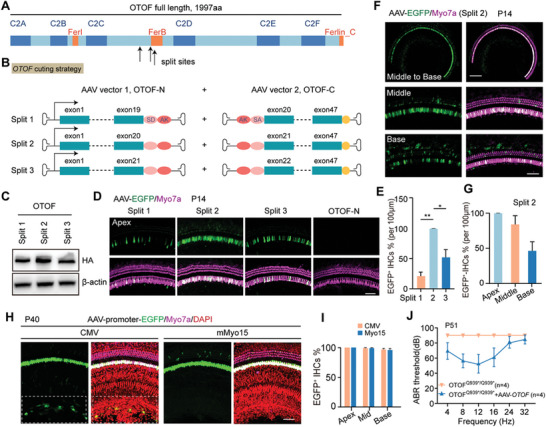
Design and construction of the gene therapy vectors. A) Human OTOF isoform one protein domains and split sites of the dual vectors. B) Schematic representation of the recombinant AAV‐vector pairs. Human otoferlin isoform one was split at exon19/20, 20/21, or 21/22, and the OTOF‐N and OTOF‐C vectors both contained AK elements for recombination. In addition, the splicing donor and splicing acceptor elements in the vectors were included for RNA splicing. C) Verification of full‐length OTOF expression in transfected HEK‐293T cells by western blot. *β*‐actin was used as the reference gene. D) Confocal images of the apical turn of the cochlea injected with AAV virus containing different split sites. The OTOF‐N virus contained a coding sequence for a separate EGFP fluorescent reporter in order to determine the recombination efficiency. The dual vectors were packaged into AAV1, and the viruses were injected into the P3 mice and the EGFP expression (green) was analyzed in Myo7a‐positive hair cells (magenta) at P14. Scale bar: 50 µm. E) The percentage of EGFP‐positive IHCs in the apical turns for split‐site 1, 2, and 3. F) Confocal images of the middle and basal turns of cochleae injected with AAV virus containing split site 2. Scale bars: 200 µm and 50 µm. G) The percentages of EGFP‐positive IHCs for spilt site 2 in the middle and basal turns. H) Confocal images of EGFP‐transduced cells in the organ of Corti under the control of the CMV and mMyo15 promoters. The P30 mice were injected with Anc80‐CMV‐EGFP or Anc80‐mMyo15‐EGFP, and immunostaining was performed at 10 days postinjection. Scale bar: 50 µm. The dashed frame indicated the greater epithelial ridge region where the CMV‐driven EGFP was expressed. I) Percentage of EGFP‐positive IHCs in the injected ears in (H). J) ABR thresholds of OTOF^p.Q939*/Q939*^ mice treated with dual AAV vectors under control of the CMV promoter (yellow, *n* = 4) or mMyo15 promoter (blue, *n* = 4). The viruses were injected at P30, and the ABR was measured 21 days postinjection. Error bars indicated the standard deviation. The *p*‐value was calculated by Student's t‐test. ^*^ *p* < 0.05, ^**^ *p* < 0.01.

The human *OTOF* coding region is 5991 bp (**Figure** **1**A), thus exceeding the packaging limit of a single AAV vector (≈4700 bp). Previous studies have shown that dual AAV strategies based on DNA trans‐splicing are effective methods for delivering long transgenes,^[^ ^27^, ^28^ ^]^ as well as mRNA trans‐splicing^[^ ^29^, ^30^ ^]^ and split intein‐mediated protein trans‐splicing,^[^ ^31^, ^32^, ^33^, ^34^, ^35^ ^]^ and the ability of both DNA and protein trans‐splicing to achieve full‐length OTOF recombination and hearing restoration varied widely.^[^ ^15^, ^16^, ^17^ ^]^ We thus supposed that the OTOF splitting site is critical for the recombination efficiency and therefore critical for the efficacy of the vector. We designed dual AAV vectors splitting between exons 19/20, 20/21, and 21/22 in *OTOF* (Figure 1B). To confirm the recombination ability of these three splitting sites, western blotting was performed to detect the expression levels of the full‐length OTOF protein, and split‐site 2 (between exons 20 and 21) showed the greatest protein expression in HEK293T cells (Figure 1C). To further confirm the recombination ability of split site 2 in cochlear hair cells, enhanced green fluorescent protein (EGFP) was tagged at the 5′ terminus of the *OTOF*‐N sequence to visualize OTOF expression.^[^ ^16^ ^]^ We injected a 1:1 mixture of AAV viruses containing the three different splits between *OTOF*‐N and *OTOF*‐C into the cochleae of postnatal day (P)2 newborn mice through the round window membrane, and we measured the proportion of EGFP‐positive IHCs after 10 days. The OTOF‐N‐EGFP vector injection group was used as a negative control to exclude interference from the EGFP background. The confocal images showed that compared with Split 1 and Split 3, the proportion of EGFP‐positive IHCs was highest in the Split 2 injection group, with almost 100% transduction efficiency in the apex of the cochlea. The EGFP expression efficiency of split 2 was more than or equal to four‐ and twofold of split 1 and split 3, respectively (Figure 1D–F). And, at least 50% of IHCs successfully expressed EGFP in the middle and basal turns of the cochlea (Figure 1G).

In mice, hearing onset occurs around P12. Whereas in humans, the development of the inner ear is completed in utero, with hearing onset at embryonic week 27 (Figure S1, Supporting Information).^[^ ^36^ ^]^ Considering that the human inner ear is fully developed during embryonic development, efforts need to be made to explore the transduction efficiency of gene therapy vectors in adult mice. Studies have shown that several different AAV serotypes can effectively transduce cochlear IHCs, such as AAV1, AAV2, Anc80L65, etc.^[^ ^18^, ^19^, ^20^ ^]^ We first verified the transduction efficiency and auditory safety of Anc80L65 and AAV1 in IHCs from both young and adult mice. Immunofluorescence staining showed that AAV1‐EGFP and Anc80L65‐EGFP had comparable transduction efficiencies in IHCs from P1‐2 mice (Figure S2A,B, Supporting Information). Although the transduction efficiency of Anc80L65 on P30 IHCs is higher than that of AAV1, both of them can successfully express exogenous transgenes in at least 80% of IHCs (Figure S2C,D, Supporting Information). These results indicate that both Anc80L65 and AAV1 can safely and efficiently transduce IHCs in adult mice and therefore can be used for the delivery of *OTOF* to IHCs.

### 2.2 OTOF Re‐Expression Driven by a Hair‐Cell‐Specific Promoter is Necessary for Long‐Term Hearing Recovery in Adult Otof Mutant Mice

To mimic the pathologic features of human DFNB9, we chose the pathological *OTOF* point mutation c.2818C>T (p.Gln940*) in ClinVar and generated a humanized mouse model by using CRISPR‐Cas9‐mediated homologous recombination to specifically introduce a *C* to *T* substitution, which converted a CAA codon to a TAA stop codon in the mouse genomic DNA. The DNA substitution in the mouse *Otof* gene yielded a p.Q939* point mutation, homologous to the p.G940* mutation in humans. Consistent with the phenotype in patients with the Otof p.G940* mutation, the ABR results showed that homozygous OTOF p.Q939* mice were completely deaf at the age of P30 (Figure S3A, Supporting Information). Two antibodies targeting the *N*‐terminus and *C*‐terminus of Otof, respectively, did not detect OTOF expression in OTOF^p.Q939*/Q939*^ mice (Figure S3B,C, Supporting Information). In addition, the number of presynaptic/postsynaptic puncta marked by Ctbp2/vGluR2 was significantly reduced (Figure S3D,E, Supporting Information). These results indicate the successful construction of the *Otof* point mutant mouse model.

It had been reported that ubiquitous promoter might lead to hearing loss in adult mice,^[^ ^25^ ^]^ so we chose the mouse hair cell‐specific promoter mMyo15 for *OTOF* gene therapy. mMyo15‐driven EGFP was selectively expressed in hair cells with almost 100% efficiency in the IHCs (Figure 1H,I), and no EGFP signals were detected in supporting cells or other cells in the cochlea (Figure 1I, Figure S4, Supporting Information). At 3 weeks after the therapeutic dual AAV delivery in P30 OTOF^p.Q939*/Q939*^ mice, the ABR results showed significant hearing recovery in mMyo15‐driven *OTOF* injected mice (Figure 1K), suggesting that the mMyo15 promoter is more suitable than the CMV promoter for *OTOF* gene therapy.

### 2.3 Pharmacodynamic Evaluation of Dual‐AAV‐OTOF Therapy

**Figure 2:**
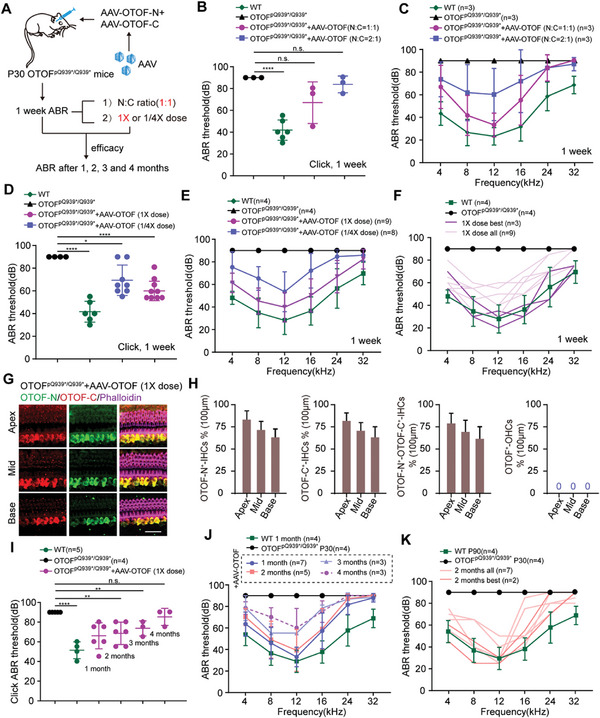
dual‐AAV‐*OTOF* injection restores auditory function in P30 OTOF^p.Q939*/Q939*^ mice. A) The experimental overview of dual AAV vector‐mediated gene therapy in adult mice. B) Click ABR thresholds of WT mice (green, *n* = 6), untreated OTOF^p.Q939*/Q939*^ mice (black, *n* = 3), and the mice treated with AAV‐*OTOF* (N:C = 1:1) (magenta, *n* = 3) and with AAV‐*OTOF* (N:C = 2:1) (blue, *n* = 3) at 1 week after injection at P30. C) Tone‐burst ABR thresholds of WT mice, untreated OTOF^p.Q939*/Q939*^ mice, and the mice treated with AAV‐*OTOF* (N:C = 1:1) and with AAV‐*OTOF* (N:C = 2:1). D) Click ABR thresholds of WT mice (green, *n* = 6), untreated OTOF^p.Q939*/Q939*^ mice (black, *n* = 4), and mice treated with AAV‐*OTOF* (1× dose = high dose) (magenta, *n* = 9) and with AAV‐*OTOF* (1/4 dose) (blue, *n* = 8). E) Tone‐burst ABR thresholds of WT mice, untreated OTOF^p.Q939*/Q939*^ mice, and the mice treated with AAV‐*OTOF* (1× dose) and with AAV‐*OTOF* (1/4 dose). F) Tone‐burst ABR thresholds of WT mice, untreated OTOF^p.Q939*/Q939*^ mice, and all the mice treated with AAV‐*OTOF* (1× dose) and the best of the 1× dose group. G) Representative confocal images of the cochlea in OTOF^p.Q939*/Q939*^ mice after injection of AAV‐*OTOF* (1× dose). Red, green, and magenta indicate *C*‐terminal OTOF, N‐terminal OTOF, and phalloidin, respectively. Scale bar: 30 µm. H) The percentages of OTOF‐N, OTOF‐C, OTOF‐N, and OTOF‐C double‐positive IHCs, and OTOF positive OHCs. I) Click ABR thresholds of WT mice, untreated OTOF^p.Q939*/Q939*^ mice, and the OTOF^p.Q939*/Q939*^ mice treated with AAV‐*OTOF* (1× dose) at 1 week, 1 month, 2 months, 3 months, and 4 months after injection at P30. J) Tone‐burst ABR thresholds of WT mice, untreated OTOF^p.Q939*/Q939*^ mice, and the OTOF^p.Q939*/Q939*^ mice treated with AAV‐*OTOF* (1× dose) at 1 week, 1 month, 2 months, 3 months, and 4 months after injection at P30. (K) Tone‐burst ABR thresholds of P90 WT mice, untreated OTOF^p.Q939*/Q939*^ mice, and all the mice treated with AAV‐*OTOF* (1× dose) and the best of the 1× dose group at 2 months after injection at P30. Error bars indicated the standard deviation. The *p*‐value was calculated by Student's t‐test. ^*^ *p* < 0.05, ^**^ *p* < 0.01, ^****^ *p* < 0.0001, and n.s. means no significant difference.

We performed an efficacy analysis of the dual AAV vectors in adult OTOF^p.Q939*/Q939*^ mice (**Figure** **2**A), including the vector ratio, vector dose, and long‐term efficacy. We injected 1:1 and 2:1 ratios (in genomic particles, GCs) of AAV‐mMyo15‐*OTOF*‐N and AAV‐*OTOF*‐C viruses into the cochleae of OTOF^p.Q939*/Q939*^ mice at P30 through the post‐semicircular canal. Auditory function was measured by ABR at 7 days after injection. The ABR results showed better hearing recovery in the 1:1 ratio of AAV‐*OTOF*‐N and AAV‐*OTOF*‐C, which came close to the hearing of wild‐type mice (Figure 2B,C). The dose of virus used is a key factor affecting therapeutic efficacy, and to obtain the optimal therapeutic dose we compared the hearing recovery abilities of a high therapeutic dose (2.8 × 10^10^ and 2.8 × 10^10^ GCs of AAV‐*OTOF*‐C, 1×dose) and a low therapeutic dose (1/4 of the high dose, 1/4×dose). ABR hearing tests on adult OTOF^p.Q939*/Q939*^ mice injected with dual AAVs for 7 days showed that both the high dose and low dose had therapeutic effects, but the high‐dose group had significantly better hearing improvement than the low‐dose group (Figure 2D,E), and ≈33% of the treated OTOF^p.Q939*/Q939*^ mice had their hearing restored to a level comparable to wild‐type mice (Figure 2F). Immunofluorescence staining showed re‐expression of *OTOF*‐N and *OTOF*‐C in the IHCs of the cochleae from the high‐dose AAV‐injected OTOF^p.Q939*/Q939*^ mice, with the rate of full‐length OTOF‐positive IHCs being as high as 80% (Figure 2G,H). While we did not detect the OTOF re‐expression in OHCs although Myo15‐promoter‐driven GFP expression is observed in OHCs (Figure 2G,H). The long‐term ABR results showed that the therapeutic effect of full‐frequency hearing recovery in adult OTOF^p.Q939*/Q939*^ mice after therapy was stable and lasted at least 2 months (Figure 2I,J), with better hearing recovery in the middle‐to‐low frequency range in some injected mice (Figure 2K). At 3–4 months after AAV treatment, the recovered hearing thresholds of OTOF^p.Q939*/Q939*^ mice deteriorated slightly, with the thresholds being elevated by ≈5–20 dB compared to 1 month after therapy (Figure 2I,J). Despite all this, hearing at low frequencies still maintained positive recovery at 4 months after injection (Figure 2J). In summary, our dual AAV strategy could effectively restore hearing in adult OTOF^p.Q939*/Q939*^mice.

### 2.4 Safety Assessment and Biodistribution of Dual‐AAV‐OTOF in Mice

To evaluate the safety and AAV vector biodistribution after a single intracochlear injection of dual AAV‐*OTOF* vectors in mice, we conducted the toxicity and biodistribution study. Therapeutic dual AAV‐*OTOF* viruses were injected into the inner ears of 4‐week‐old C57BL/6J wide‐type mice using the same surgical approach as gene therapy. Changes in auditory function, motor ability, learning and memory abilities, histopathology, and clinical pathology were evaluated at 1 week, 2 weeks, and 2 months after surgery, and neutralizing antibodies in serum and AAV biodistribution were explored. To ensure the accuracy of the results, the body weights of all animals of the same gender in each group were within ±20% of the mean body weight.

**Figure 3:**
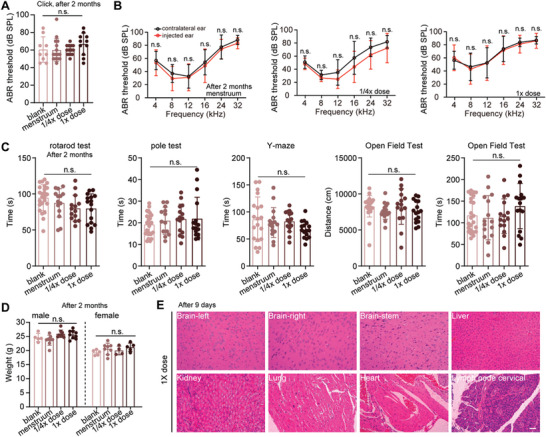
Dual AAV‐OTOF delivery has minimal adverse effects in WT mice at 2 months after injection at P30. A) Click ABR of adult wild‐type mice and wild‐type mice injected with menstruum, 1/4 dose, and 1× dose of AAV‐OTOF. B) ABR of wild‐type mice injected with menstruum and virus. C) The behavior tests of adult wild‐type mice and wild‐type mice injected with menstruum and virus. D) The body weight of adult wild‐type mice and wild‐type mice injected with menstruum and virus. E) The HE staining of multiple organs from wild‐type mice injected with the virus. Scale bar: 50 µm. Error bars indicated the standard deviation. The *p*‐value was calculated by Student's *t*‐test or one‐way ANOVA. n.s. means no significant difference.

To verify whether dual‐AAV‐mMyo15‐*OTOF* affects mouse hearing, ABR testing was performed on the menstruum‐injected mice (menstruum refers to the solvent used to dissolve AAV‐*OTOF*‐N and AAV‐*OTOF*‐C), the 1× dose (2.8 × 10^10^ vg of AAV‐*OTOF*‐N and 2.8 × 10^10^ vg of AAV‐*OTOF*‐C), and the 1/4× dose injected mice at 1 week, 2 weeks, and 2 months after injection to evaluate short‐term and long‐term hearing function. The results showed no significant change in ABR thresholds across frequencies in the menstruum, low dose, and high dose groups compared with the contralateral ears (**Figure** **3**A,B; Figure S5A,B, Supporting Information), indicating that surgery and dual‐AAV‐*OTOF* did not affect normal auditory function in mice. Next, we evaluated the motor and memory abilities of injected mice 1 month after injection by using the rotarod test, pole test, *Y*‐maze test, and open field test. These results showed no difference in exploratory abilities, cognitive abilities, or learning and memory functions among the menstruum, low‐dose, and high‐dose mice and the blank controls at the same age (Figure 3C). We also measured the body weights of male and female mice in all groups, and no significant differences in body weights were seen at 2 weeks or at 2 months (Figure 3D; Figure S5C, Supporting Information). In addition, we detected the neutralizing antibodies against Anc80L65 in serum at 7 weeks after injection. No neutralizing antibodies were detected in any animals in the blank or menstruum controls of the same age at different time points after injection. The titers of neutralizing antibodies against AAV‐*OTOF*‐N and AAV‐*OTOF*‐C in the low and high dose groups at 7 weeks after injection were 1:10–1:160 and 1:80–1:640, respectively (Figure S4D, Supporting Information), which indicated that dual AAV‐*OTOF* injection could induce neutralizing antibodies against AAV‐*OTOF*, with overall higher titers in the high dose group.

Before euthanizing the mice, we determined the genomic DNA (gDNA) biodistribution of the dual AAV vectors in blood and major organs. The details are shown in Table S1 (Supporting Information). At 9 days after injection, the detection ranges of *OTOF*‐N in the left cochleae of the low and high dose groups were 1.31 × 10^4^ –7.22 × 10^4^ and 4.34 × 10^3^ –5.88 × 10^4^ copies µg^−1^ gDNA, respectively, and the detection ranges of *OTOF*‐C in the cochleae of the low and high dose groups were 1.52 × 10^4^–6.38 × 10^4^ and 4.11 × 10^3^–7.20 × 10^4^ copies µg^−1^ gDNA, respectively, indicating the rapid and abundant distribution of AAV‐*OTOF*‐N and AAV‐*OTOF*‐C at the injection site. At this time, AAV‐*OTOF*‐N and AAV‐*OTOF*‐C were also detected in blood and other tissues, and higher copies of dual vectors were detected in the liver compared to other organs, with the maximum variation being more than a thousandfold change. The distribution of dual vector DNA in the liver was comparable to that in the cochlea and showed a dose dependency. The above results indicate that AAV5‐*OTOF*‐N and AAV‐*OTOF*‐C enter the circulatory system after inner ear injection and have a higher distribution in the liver.

Because AAV could be detected in many tissues, we evaluated the histopathological changes of the major organs after AAV injection. Mice were sacrificed 9 days after surgery, and hematoxylin‐eosin (HE) staining was used to observe histopathological changes. Microscopic examination of HE‐stained tissue sections from most of the menstruum, low‐dose, and high‐dose groups showed no abnormal pathological changes (Figure 3E). In addition, whole blood was collected and analyzed presurgery and at 9 days postsurgery. No significant differences were detected in the blood cell counts or biochemical parameters. The changes in individual indexes were not significantly different (Tables S2,S3, Supporting Information). In summary, dual AAV‐Otof injection caused no abnormal changes in body weight, blood profiles, auditory function, motor/memory functions, or histopathology, thus indicating the safety of therapeutic doses of AAVs in mice.

### 2.5 Establishment of a Safe Method for Inner Ear Drug Delivery in NHPs

Round window injection is an effective method for AAV delivery. The round window membrane can be exposed through the trans‐mastoid‐facial recess or trans‐external canal, but cynomolgus monkeys have a narrow external auditory canal that is difficult to access with an otoscope, and they also have the disadvantage of postoperative atresia in the ear canal. Therefore, we exposed the round window membrane through the trans‐mastoid‐facial recess approach.

**Figure 4:**
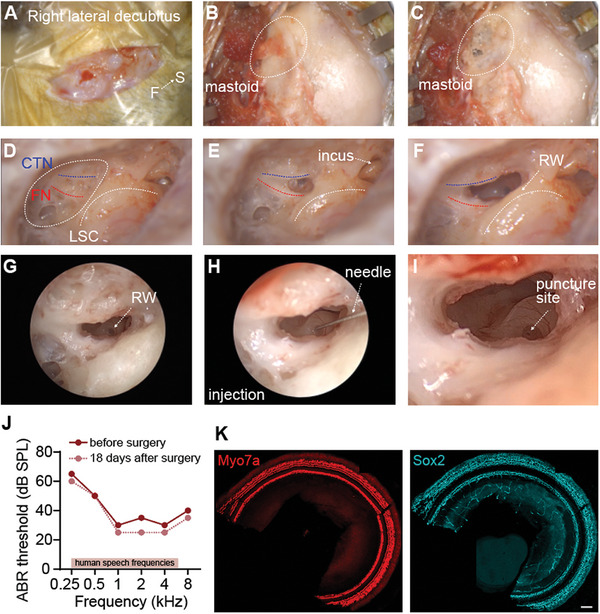
Transmastoidal exposure of the round window for AAV injection into the cochlea of cynomolgus monkeys. The procedure shows the round window exposure for AAV injection in the right ear. A posterior incision was made in the auricula of the right ear. A) and the subcutaneous muscle was peeled back to expose the mastoid indicated by the dashed ring. B) The mastoid was carefully polished using an ontological microdrill to expose the chorda tympani nerve (CTN, blue line), facial nerve (FN, red line), and lateral semicircular canal (LSC, white line). C–E) Here, the incus can be visualized E). The round window was located below the angle between the CTN and FN. F) AAV was delivered by puncturing the round window membrane with a 33G needle G–I) ABR. J) and immunofluorescence staining K) was performed 18 days after surgery showing Myo7a‐labeled hair cells (red) and Sox2‐labeled supporting cells (cyan). Scale bar: 100 µm.

During surgery, an arc‐shaped incision of ≈3 cm was made behind the auricle 0.5 cm away from the retroauricular sulcus. After incising the skin and periosteum, the mastoid was exposed. The mastoid area, the posterior lateral wall of the epitympanum, and the mastoid antrum were resected under the microscope by using a high‐speed electric drill to expose anatomical landmarks including the sigmoid sinus, sinus tympani, tegmen tympani, horizontal semicircular canal, short process of incus, incudomalleolar joint, etc. The mastoid antrum below the horizontal semicircular canal was contoured. The vertical segment of the facial nerve and chorda tympani nerve were delineated, and the facial recess was opened to access the posterior tympanum, thus exposing the tendon of the stapedius muscle and the incudostapedial joint, and then fully exposing the round window membrane. Local injection was performed through the round window membrane using an ear endoscope (**Figure** **4**A). Immunofluorescence and ABR were used to evaluate the effects of the round window injection on hearing and cochlear structure in cynomolgus monkeys. There was no obvious loss of cochlear hair cells or supporting cells in cynomolgus monkeys, and there were no significant changes in hearing at speech‐related frequencies after surgery (Figure 4B,C). The above data indicate that round window injection via the trans‐mastoid‐facial recess approach is a safe method for inner ear drug delivery.

### 2.6 Dual‐AAV‐OTOF Efficiently Transduces IHCs in NHPs

**Figure 5:**
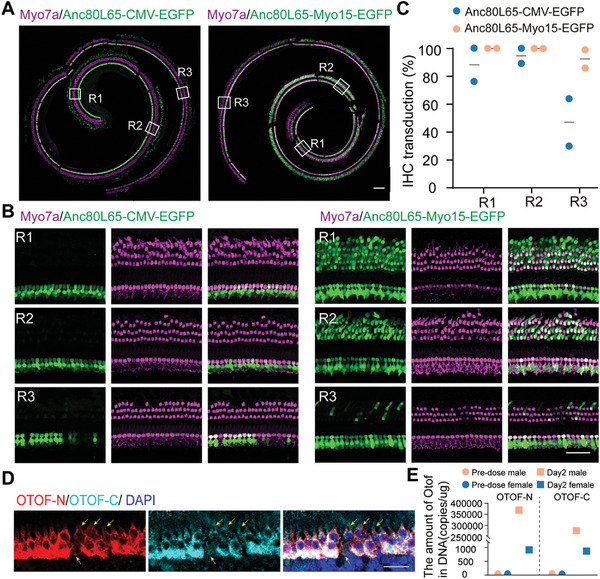
mMyo15‐driven EGFP via Anc80L65 was expressed in OHCs and IHCs in cynomolgus monkeys. A) Representative confocal images of cochlear spirals staining for Myo7a (magenta) and EGFP (green) after transduction with Anc80L65‐CMV‐EGFP and Anc80L65‐mMyo15‐EGFP, respectively. Scale bar, 200 µm. B) Magnified regions from A), indicated by R1, R2, and R3. Scale bar, 50 µm. C) The IHC transduction rates of Anc80L65‐CMV‐EGFP and Anc80L65‐mMyo15‐EGFP in different cochlear regions. Two ears in each group were used in the calculation. D) Representative confocal images of IHCs transduced with Anc80L65‐mMyo15‐*OTOF*‐N and Anc80L65‐*OTOF*‐C. Arrows indicate the un‐transduced IHCs. Z‐stack images of IHCs were captured and projected. Scale bar, 30 µm. E) Quantification of genomic DNA of mMyo15‐driven *OTOF*‐N and *OTOF*‐C in the blood. Blood samples were collected from four cynomolgus monkeys of both sexes after AAV injection for 2 days.

By using the established round window drug delivery approach in cynomolgus monkeys, we first clarified the IHC transduction efficiency of AAV‐mMyo15‐EGFP. Immunofluorescence imaging showed that the mMyo15 promoter could drive EGFP expression in cynomolgus monkey IHCs with a higher transduction rate compared with the CMV promoter (**Figure** **5**A–C). In detail, the percentage of IHCs expressed with mMyo15‐driven EGFP was comparable with that in the CMV groups in the apical turn of the cochlea. While in the middle to basal turns, the IHC transduction rates of mMyo15‐EGFP were relatively high with 1.5–3 folds of that in CMV (Figure 5C). Surprisingly, mMyo15‐driven EGFP was abundantly detected in outer hair cells (OHCs) with almost 100% in the apical to middle turns, while CMV (Figure 5B) and CB7^[^ ^21^ ^]^ could not drive EGFP expression in OHCs. Neither the mMyo15 nor the CMV promoter had expression activity in supporting cells (Figure S6A, Supporting Information). In addition, CMV could drive EGFP expression in the lesser epithelial ridge cells lateral to the organ of Corti, while mMyo15 was only expressed in the hair cells (Figure S6B, Supporting Information), indicating that AAV‐mMyo15‐EGFP specifically targets hair cells. These experiments demonstrate that the mMyo15 promoter specifically and effectively drives transgene expression in cynomolgus monkey cochlear hair cells.

Next, we injected AAV‐*OTOF*‐N and AAV‐*OTOF*‐C at a 1:1 ratio into cynomolgus monkey cochleae and measured the *OTOF* expression in hair cells 4 months later. Immunofluorescence signals showed variable intensities of OTOF‐N and OTOF‐C within the adjacent IHCs (Figure 5D), and the relative intensity of OTOF‐N was comparable with that of OTOF‐C (Figure 5D). We did not find immunosignals in OHCs (Figure S7, Supporting Information), which was consistent with the result in mice (Figure 2G). However we detected genomic *OTOF*‐N and *OTOF*‐C in the blood, and the results indicated that the AAVs rapidly entered the circulatory system after injection and the number of genomic AAV‐*OTOF*‐N and AAV*‐OTOF*‐N was comparable within each other (Figure 5E), indicating a similar dose administration of the two AAVs. Taken together, these results demonstrate that the mMyo15 promoter can effectively drive exogenous *OTOF* expression in the IHCs of cynomolgus monkeys.

### 2.7 Safety Assessment of Dual‐AAV‐OTOF in NHP

**Figure 6:**
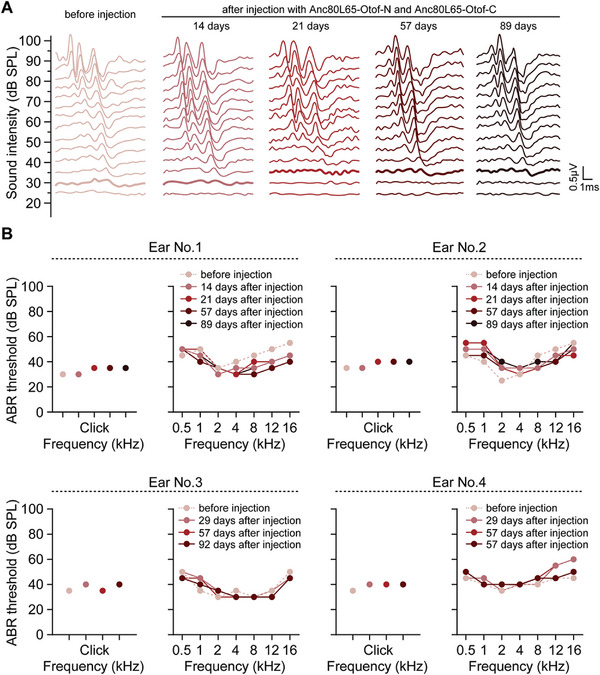
Dual‐AAV‐*OTOF* injection did not affect normal hearing in cynomolgus monkeys. A) Representative ABR traces were recorded for Click stimuli from a cynomolgus monkey (No. 1 in panel B) injected with mixed AAV‐*OTOF*‐N (4.2 × 10^11^ GCs) and AAV‐*OTOF*‐C (4.2 × 10^11^ GCs). Colored lines indicate the hearing thresholds. B) Individual ABR results from four ears in response to pure tones of different frequencies (0.5, 1, 2, 4, 8, 12, and 16 kHz) before and after injection at different time points.

We next evaluated the short‐term and long‐term auditory safety of dual‐AAV‐*OTOF* delivery into the cynomolgus monkey inner ear. We first selected two cynomolgus monkeys ≈4 years old with normal hearing for surgery. The surgery date is defined as day one. The dual‐AAV‐*OTOF* mixture was injected into both the left and right ears of each cynomolgus monkey. Closed‐field ABR tests were then performed on each ear at different times after AAV injection. The ABR results showed no significant difference in the hearing thresholds of click or pure tones of different frequencies between pre‐injection and 14 days, 21 days, 29 days, 57 days, and 89–92 days postinjection in the same ear (**Figure** **6**A,B), indicating that the surgical procedure and dual‐AAV‐*OTOF* have no adverse effect on normal auditory function and have long‐term auditory safety in cynomolgus monkeys.

**Figure 7:**
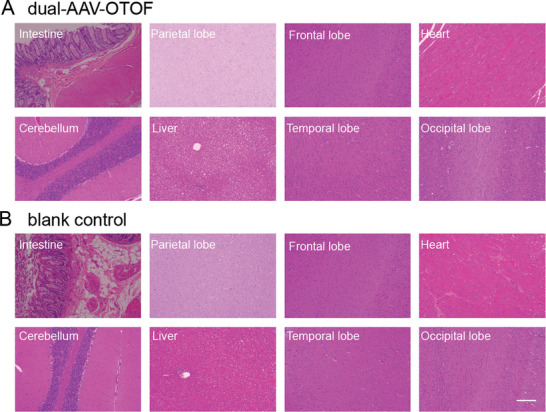
HE staining of tissues from cynomolgus monkeys. The HE staining of multiple tissues from cynomolgus monkeys injected with mixed AAV‐*OTOF*‐N (4.2 × 10^11^ GCs) and AAV‐*OTOF*‐C (4.2 × 10^11^ GCs) A) and blank controls B). Scale bar: 200 µm.

To assess the potential systemic toxicity of this dual‐AAV‐*OTOF* strategy in NHPs, we measured the systemic toxicity and inflammatory responses after AAV injection. Blood samples were collected and compared to blank cynomolgus monkey controls at 4 months after AAV injection. Although we observed changes in some individual parameters, these changes were not clinically significant (Table S4, Supporting Information). Also, the histopathological changes were evaluated in the major organs after AAV injection. No morphological or pathological changes were detected in the brain, spinal cord, heart, liver, kidney, intestine, etc., stained with HE 4 months after AAV injection (**Figure** **7**). In summary, there were no abnormal changes in blood biochemistry or histopathology after AAV injection in cynomolgus monkeys. Combined with the hearing test results, these findings suggest that dual‐AAV‐*OTOF* is safe for clinical use.

## 3 Discussion

Although some progress has been made in the research into *OTOF*‐related gene therapy, questions remain regarding product design, efficacy, and safety. Through screening and comparison, we found that Anc80L65 had higher transduction efficiency in IHCs of adult mice compared to other serotypes. A comparison of promoters revealed that the hair cell‐specific promoter mMyo15 could stably restore hearing in deaf OTOF^p.Q939*/Q939*^ mice for a longer time than the CMV promoter. We also compared different splitting sites of *OTOF* for dual AAV vector construction. Ultimately, we found that AAV‐mMyo15‐*OTOF* with the splitting site between exon 20 and 21 could stably and persistently restore hearing in adult OTOF^p.Q939*/Q939*^ mice. Moreover, injection of the dual‐AAV‐*OTOF* mixture did not affect hearing or induce systemic toxicity in adult mice or NHPs. The biodistribution of dual‐AAV‐*OTOF* viruses showed accumulation in organs like the liver after local inner ear injection, indicating that AAV tropism for the liver might be a safety issue in hearing gene therapy. Therefore, the efficacy and safety data in our study provide systematic information for subsequent clinical trials in humans.

We have restored the hearing function of mouse models after adult injection, but the effects are slightly decreased. Other studies could restore hearing to WT level for the long‐term after neonatal injection.^[^ ^15^, ^17^ ^]^ This difference may be because of the exogenous expression level of OTOF and the AAV targeting efficiency. With the same AAV serotype and dose, neonatal injection always leads to a higher transduction rate and expression of transgene than adult injection in the cochlea. The other reason for progressive hearing loss in aging mice might result from *OTOF* transcript alterations. There are five annotated *Otof* isoforms, and recently, Liu et al.^[^ ^37^ ^]^ identified multiple transcripts of the *Otof* gene and other genes. They found that one truncated transcript could restore the hearing of *Otof* knock‐out mice. And our data found that annotated isoform five could rescue hearing function in mouse models. However, the function and the expression patterns of the other isoforms remain largely unknown, and it remains uncertain whether progressive hearing loss in aging mice results from transcript alterations of *OTOF* or other genes. More experiments, such as single‐cell sequencing and AAV‐mediated delivery of specific isoforms, could be performed to elucidate the transcript alterations of deafness genes in the aging process.

Currently, *OTOF*‐related gene therapy products from two companies, Akouos and Decibel, have entered investigational new drug (IND) stages. Based on public information, these therapies use Anc80L65 and AAV1 vectors, respectively, both of which are commonly used vectors in hearing gene therapy. ^[^ ^11^, ^13^, ^38^, ^39^ ^]^ AAV1 has lower hair cell transduction efficiency in adult mice (Figure S2, Supporting Information) and cynomolgus monkey ^[^ ^21^ ^]^ hair cells than Anc80L65, and we also verified the transduction efficiency of Anc80L65 in cynomolgus monkeys, which was similar to the published data.^[^ ^21^ ^]^ Thus, we selected Anc80L65 as the delivery vector for our preclinical study. However, it is worth noting that we observed Anc80‐OTOF distribution in the brain and liver (Table S1, Supporting Information), indicating AAV's potential risks to other organs through inner ear local delivery. Although we did not observe any histopathological lesions in mice or cynomolgus monkeys, our observation period was relatively short and the long‐term risks remain unclear. Therefore, developing more specific AAV vectors in order to reduce transduction of other tissues and using hair cell‐specific promoters to reduce transgene expression in off‐target cells are important for hearing gene therapy.

The ubiquitous promoter CMV showed retinal cell damage after local eye injection,^[^ ^40^ ^]^ and another ubiquitous promoter—CAG—was also found to damage hearing in the adult mouse cochlea, while the CMV promoter was not ototoxic.^[^ ^25^ ^]^ Strangely, our results showed that CMV‐driven *OTOF* could only restore hearing at 7 days postinjection, and this recovery could not be maintained long‐term. In contrast, the hair cell‐specific promoter mMyo15 that we used from patent US20210388045A1 could stably maintain long‐term hearing recovery. These results indicate that even if the promoter itself may be safe, it may have some negative effects when combined with the transgene cassette.

There are currently two therapeutic strategies for large genes, namely splice donor‐splice acceptor (SD–SA)‐mediated trans‐splicing ^[^ ^15^, ^16^ ^]^ and intein‐mediated protein trans‐splicing.^[^ ^17^ ^]^ Both strategies can achieve good hearing recovery in neonatal mice, but intein‐mediated protein splicing has been shown to be less effective than SD‐SA‐mediated splicing in adult *Otof* knockout mice.^[^ ^17^ ^]^ The reasons for this difference may involve the *OTOF* isoform, the promoter, or the *OTOF* split site. Considering the therapeutic time window, we selected the SD‐SA‐mediated trans‐splicing approach for dual vector delivery of the *OTOF* gene in adult mice. Unlike the previous two studies using mouse *OTOF* sequences,^[^ ^15^, ^16^ ^]^ this study delivered the human *OTOF* gene and analyzed several splitting sites, and found that splitting at exons 20 and 21 achieved higher full‐length OTOF expression (Figure 1). We achieved similar effects to Akil's study^[^ ^15^ ^]^ with better recovery than Al‐Moyed's study.^[^ ^16^ ^]^ In addition, hearing recovery in adult mice was better than that with intein‐mediated gene therapy.^[^ ^17^ ^]^

In summary, this study optimized various aspects of dual AAV‐*OTOF* vectors for use in DFNB9‐related gene therapy, thus providing a preclinical drug design strategy with good efficacy in adult mice and verifying its safety in both mice and cynomolgus monkeys. However, we did find that AAV was more broadly distributed in other tissues after local delivery in the inner ear. Although no significant toxicity was observed over the 2‐month observation period, developing more specific AAV serotypes and promoters remains important for evaluating the long‐term safety of hearing‐related gene therapy.

## 4 Experimental Section

### Animals

C57BL/6 mice and OTOF‐p.Q939* mice of both sexes were used in this research. OTOF‐p.Q939* transgenic mice were obtained from a C57BL/6J mouse model with a point mutation (p.Q939*) in the mouse *Otof* locus using CRISPR/Cas‐mediated genome engineering. The day of birth was determined as P0. The genotyping primers for OTOF‐p.Q939* mice were Forward, 5′‐TAT CTA CCT CTG CCT AAA GCT TCA AC‐3′, and Reverse, 5′‐GCT CTG GTT GAT GAA GAA GAC AC‐3′. Animals were housed under a 12 h light/dark cycle at 22 ± 1 °C with food and water available ad libitum. All experiments were approved by the Institutional Animal Care and Use Committee of Southeast University, China (experiment number: 20 210 606 001).

Cynomolgus monkeys were raised and treated in accordance with the standard operating procedure of JOINN Laboratories (Suzhou) Co., Ltd. and Nanjing ClinBridge Biotech Co., Ltd. All experimental procedures were approved by the Animal Care and Use Committee (IACUC) at JOINN Laboratories (Suzhou) Co., Ltd. (experiment number: S‐ACU23‐0663) and Nanjing ClinBridge Biotech Co., Ltd. (experiment number: AP‐C220602 and AP‐C220602‐20230609). Animals 3–7 years old of both sexes were used in this study. Single animals were housed in stainless steel cages. Animals were kept in a well‐ventilated facility with controlled environmental conditions. The room temperature of the animal house was controlled at 18‐26 °C, relative humidity was controlled at 40%−70%, and light illumination was alternated between light and dark every 12 h.

### Viral Vectors

AAV was constructed with serotype AAV1 or Anc80L65. The transgenes contained within the viruses were CMV‐*EGFP*, mMyo15‐*EGFP*, CMV‐*OTOF*‐N, *OTOF*‐C, mMyo15‐*OTOF*‐N, and *OTOF*‐C. Here, the coding region of *OTOF* was split into two parts, *OTOF*‐N and *OTOF*‐C, with splice sites designed between exons 19/20, 20/21, and 21/22. The *OTOF*‐N plasmid contained the N‐terminus of *OTOF* and a splice donor, while the *OTOF*‐C plasmid contained a splice acceptor and the C‐terminal of *OTOF*. CMV or mMyo15 promoters were used to drive transgene expression. In some experiments, EGFP or HA tag proteins were inserted at the 5′ end or 3′ end of the coding sequences of *OTOF*‐N or *OTOF*‐C. Detailed procedures for AAV packaging and titration can be found in our previously published paper.^[^ ^41^ ^]^ All viruses were packaged using a triple‐plasmid system, including capsid (AAV1 or Anc80L65) plasmid, helper plasmid, and transgene plasmid.

### AAV Injection in Mice

For P1‐3 neonatal mice, anesthesia was induced by an ice‐water bath. A postauricular incision was made and the round window niche was exposed. AAV viruses were loaded into a glass electrode with fine tips, and 1–2 µl AAV per ear was injected through the round window membrane. The wound was glued with tissue adhesive (Vetbond, 1469SB) and the mouse pups were placed on a 38 °C heating pad for recovery before being returned to their mother.

For adult mice, anesthesia was induced by intraperitoneal injection of tribromoethanol (500 mg kg^−1^). The left postauricular area was shaved, and an incision was made to expose the posterior semicircular canal. A hole was poked into the posterior semicircular canal, and 1–2 µl of AAV was slowly injected through the hole. Preprepared fat was used to quickly seal the hole, and the incised skin was glued with tissue adhesive (Vetbond, 1469SB). Mice were placed on a 38 °C heating pad for recovery after surgery. Mice were also injected with the solvent used to dissolve AAV‐OTOF‐N and AAV‐OTOF‐C, referring to to the menstruum group.

### Immunofluorescence

Cochleae were dissected in cold PBS and fixed in 4% PFA for 2–24 h then decalcified in 0.5 m EDTA and dissected into pieces before being transferred to Cell‐Tak (Corning, 354 240)‐coated slides. Cochlear samples were blocked in a medium containing 10% donkey serum and 0.5% Triton‐X100 in PBS at room temperature for 1–2 h. Primary antibodies against Myosin7a (Proteus Bioscience, 256 790), OTOF‐N (Abcam, ab53233), OTOF‐C (Thermo fisher, PA552935), Sox2 (R&D systems, AF2018), Ctbp2 (Biosciences, 612 044), and GluR2 (Millipore, MAB397) were added and incubated overnight at 4 °C. The next day the primary antibodies were removed and the cochleae were washed three times for 3 min each with 0.1% Triton‐X100 solution. Secondary antibodies were incubated at room temperature for 1 h. Samples were then mounted in a fluorescence mounting medium (DAKO, S3023) and imaged with a confocal microscope (Zeiss, LSM900).

### ABR Measurement in Mice

Mice were anesthetized with tribromoethanol (500 mg kg^−1^), and ABR thresholds in response to click and pure tone bursts (4, 8, 12, 16, 24, and 32 kHz) were measured to evaluate hearing sensitivity. During the recording, anesthetized mice were placed on a 37 °C heating pad. Needle electrodes were inserted subdermally at the vertex and mastoid portion, with a ground electrode was inserted at the thigh. ABRs were recorded using the TDT RZ6 auditory workstation (Tucker‐Davis Technologies, USA), and thresholds were determined by the lowest stimulus that elicited repeatable ABR responses. Body temperature was held constant throughout the experiment.

### Scanning Electron Microscope Imaging

Cochleae were dissected in cold HBSS and fixed by perfusion of 2.5% glutaraldehyde through a hole in the apex of the cochlea. Cochleae were decalcified in 0.5 M EDTA for 12 h and dissected into pieces. Cochlear samples were then post‐fixed in 2% osmium tetroxide solution at 4°°C for 2–4 h, washed in distilled water, immersed in 1% thiocarbohydrazide solution two times for 30 min each, and dehydrated in an ethanol series (50%, 70%, 90%, 100%) for 10 min each time. Samples were critical point dried, sputter coated with 10 nm of platinum (JEC‐3000FC, JEOL Ltd.), and imaged with a scanning electron microscope (JSM‐7800F prime, JEOL Ltd.).

### Behavioral Tests in Mice

*Rotarod Test*: Mice were placed on a rotarod apparatus set to accelerate from 10 to 40 rpm min^−1^ as follows: blanking speed of 10 rpm min^−1^, acceleration time of 6 s, duration of 30 s; initial speed of 25 rpm min^−1^, acceleration time of 8 s, duration of 30 s; middle speed of 40 rpm min^−1^, acceleration time of 8 s, duration of 30 s; and maximum speed of 50 rpm min^−1^, acceleration time of 6 s, duration of 30 s. The time points, speeds, and distances at which the mice fell were recorded. Each mouse underwent three trials with a 5 min inter‐trial interval. The device was cleaned and sterilized between each animal tested.

*Pole Test*: Mice traversed a 1 m long wooden pole elevated at a 30° incline ending in a box. During the experimental procedure, the mice were placed head down onto a ball at the tip of the pole, and the time was recorded from when the mice touched the ball to when the mice landed on the ground with their front paws. If the mice stopped in the middle of the test or crawled in the opposite direction, the results were not recorded, and the test was repeated. Each mouse underwent two trials with a 4 h inter‐trial interval. Results from the two trials were averaged.

*Open Field Test*: Mice were placed in the center of a 50 × 50 × 40 cm open field chamber and allowed to explore freely for 30 min. Activity was monitored by a video camera. The total distance traveled, and the time spent being mobile were analyzed using video tracking software. The chamber was cleaned with 75% ethanol between trials.

Y‐*Maze* Test: The *Y*‐maze apparatus has three enclosed arms (35 cm length, 10 cm width, 20 cm height) diverging at 120°. Food was placed at the end of one arm. Mice were placed at the start of one arm and allowed to explore freely to find the food. The path taken and the time each mouse took to find the food was recorded. The maze was cleaned with 75% ethanol between trials.

### Hematoxylin‐Eosin Staining

Samples were fixed in 4% PFA, decalcified in 0.5 m EDTA at room temperature, and dehydrated in an ethanol series (30%, 50%, 70%, 80%, 90%, 95%, and 100%). Tissues were cleared in xylene, embedded in paraffin, sectioned at 8–10 µm, and stained with hematoxylin‐eosin solution. Images of different tissues were captured using a Leica fluorescent microscope (Leica, DMI).

### Inner Ear Injection in Cynomolgus Monkeys

Cynomolgus monkeys were anesthetized with propofol (5 mg kg^−1^ min^−1^) for intubation and maintained at 0.1‐0.5 mg kg^−1^ min^−1^. Vital signs including respiration, heart rate, blood pressure, and blood oxygen saturation were monitored throughout the surgery. Supplemental doses of propofol or ketamine (4‐12 mg kg^−1^ at 50 mg mL^−1^) were given as needed. A post‐auricular incision was made to expose the round window niche for injection (see Figure 4 for details). AAV was injected through the round window membrane using a 33G needle at a speed of 20–100 nL s^−1^ and a total viral volume of 20–40 µL. The surgical site was sutured closed after injection. Postoperative analgesics and antibiotics were administrated, including cefazolin for 7 days and meloxicam for 5 days. Animal activity, diet, wound healing, and stool were observed daily following surgery.

### ABR Measurement in Cynomolgus Monkeys

Cynomolgus monkeys were anesthetized with propofol (5 mg kg^−1^ min^−1^) and maintained at 0.1‐0.5 mg kg^−1^ min^−1^ for ABR recording. Needle electrodes were placed subdermally at the vertex and in the ipsilateral mastoid portion, with a ground electrode at the contralateral mastoid portion. ABRs were recorded using a Neuro‐Audio auditory evoked potential workstation (Neurosoft) in response to click and pure tone burst stimuli (0.5, 1, 2, 4, 8, 12, and 16 kHz) from 20 dB SPL to 90 dB SPL to determine the hearing threshold. The body temperature was held constant throughout the recording.

### Tissue DNA Biodistribution

Approximate 20 mg of tissue was homogenized and lysed using cell lysis buffer (MagMAX DNA Cell and Tissue Extraction Buffer) supplemented with RNase A and proteinase K. DNA was extracted using the MagMAX DNA Multi‐Sample Ultra 2.0 Kit (Thermo Fisher) according to the manufacturer's protocol. Tissue DNA samples were analyzed by qPCR using standard curves generated with dual‐vector plasmids. Primers/probes for *OTOF*‐N: Forward: 5′‐ GGA GCT GGT AAG TAT CAA GGT‐ 3′; Reverse: 5′‐GAA ATC GGC AAA ATC CCA GA‐ 3′; Probe: FAM‐ ACT GGG CTT GTC GAG ACA GAG AAG ACT‐ BHQ1. Primers/probe for *OTOF*‐C: Forward: 5′‐ TAC AAT TCA CGC GTG CTA GC‐ 3′; Reverse: 5′‐ TCC TGT GGA GAG AAA GGC AA‐3′; Probe: FAM‐ GCA CCT ATT GGT CTT ACT GAC‐ MGB. qPCR reactions were run on a Real‐Time PCR System (QuantStudio Design) to determine the DNA copies based on the standard curves.

### Statistical Analysis

Statistical data were collected and analyzed in GraphPad Prism 6.07 (GraphPad Software, Inc.). Two‐tailed unpaired Student's *t*‐test or one‐way ANOVA was used to determine the statistical significance of differences. A value of *p* < 0.05 was considered statistically significant.

## Conflict of Interest

C.Y., L.J., J.L., L.W., and Z.Z. are paid employees of Otovia Therapeutics Inc. Si.S. is paid employee of Otovia Therapeutics Inc. and Fosun Health Capital.

## Author Contributions

J.Q., L.Z., F.T., and Y.Z. contributed equally to this work. Conceptualization: R.C., Q.W., J.Q., F.T., L.L, and S.S. conceived and designed the experiments. Investigation: J.Q., L.Z., and F.T. performed most of the experiments except that L.L. performed the AAV injection in NHPs. Experiment assistance: Y.Z., Z.Z., C.Y., and L.J. helped with the experiments regarding the safety assessment in mice and the surgery for AAV injection in NHPs. Data analyses: J.Q. and L.Z. analyzed and presented the data. S.Z., Si.S., and H.W. helped with the data interpretation and presentation. Manuscript preparations: R.C., J.Q., L.Z., and F.T. discussed the data analysis, interpretation, and presentation and wrote the manuscript with contributions from all authors.

## Supporting information

Supporting Information

## Data Availability

The data that support the findings of this study are available in the supporting information of this article.
