# Correction to “Preclinical Efficacy and Safety Evaluation of AAV‐OTOF in DFNB9 Mouse Model and Nonhuman Primate”

**DOI:** 10.1002/advs.202416185

**Published:** 2025-01-10

Jieyu Qi, Liyan Zhang, Fangzhi Tan, Yang Zhang, Yinyi Zhou, Ziyu Zhang, Hongyang Wang, Chaorong Yu, Lulu Jiang, Jiancheng Liu, Tian Chen, Lianqiu Wu, Shanzhong Zhang, Sijie Sun, Shan Sun, Ling Lu, Qiuju Wang, and Renjie Chai. *Adv Sci (Weinh). 2024 Jan;11(3):e2306201*.

https://doi.org/10.1002/advs.202306201

In the original published paper, we found that the HE image of the frontal lobe in the blank control group in Figure 7 was mistakenly duplicated from the dual‐AAV‐OTOF group. The corrected figure is shown below. This correction does not affect the overall findings and conclusions of this paper.

Corrected Figure 7:


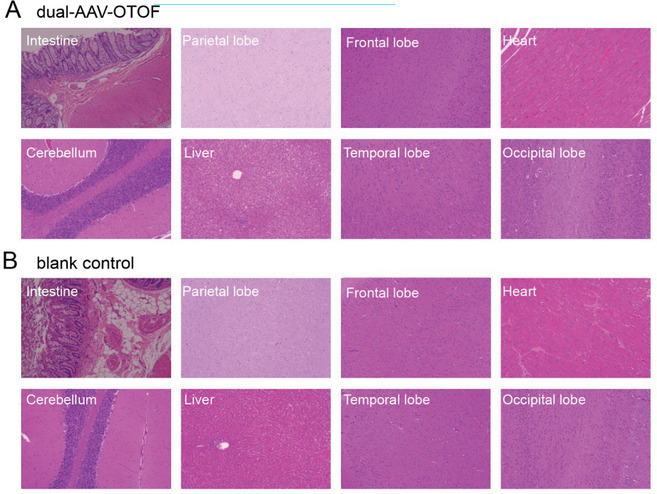


We apologize for this error.

