# Supplementary material for: Preclinical Efficacy And Safety Evaluation of AAV‐OTOF in DFNB9 Mouse Model And Nonhuman Primate
Source: Adv Sci (Weinh). 2023 Nov 28;11(3):2306201. doi: 10.1002/advs.202306201 (PMC10797419; doi:10.1002/advs.202306201)
Supplement: Supplementary file 1 — Supporting Information [file ADVS-11-2306201-s001.pdf]

ADVANCED SCIENCE
Open Access

Supporting Information

for *Adv. Sci.*, DOI 10.1002/advs.202306201

Preclinical Efficacy And Safety Evaluation of AAV-*OTOF* in DFNB9 Mouse Model And Nonhuman Primate

Jieyu Qi, Liyan Zhang, Fangzhi Tan*, Yang Zhang, Yinyi Zhou, Ziyu Zhang, Hongyang Wang, Chaorong Yu, Lulu Jiang, Jiancheng Liu, Tian Chen, Lianqiu Wu, Shanzhong Zhang, Sijie Sun, Shan Sun*, Ling Lu*, Qiuju Wang* and Renjie Chai**

Supplementary materials for

Pre-clinical efficacy and safety evaluation of AAV-*OTOF* in DFNB9 mouse model and non-human primate

This file included:

Figure S1-S7

Table S1-S5

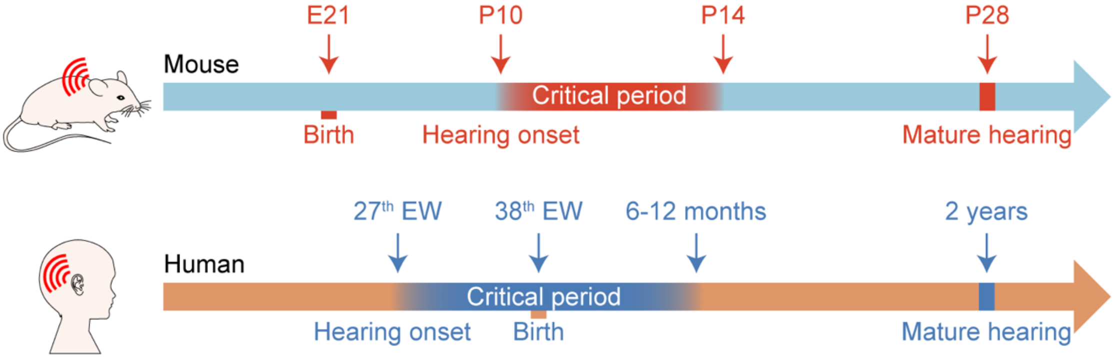

Figure S1. **The hearing development period in mouse and human.** In mice, hearing onset occurs around postnatal day 12 (P12). Whereas in humans, the development of inner ear is completed in utero, with hearing onset at embryonic week (EW) 27. Here, the injection time for studying the efficacy in mice was P30, corresponding to the age of human being 2 years after birth, indicating the treatment time window.

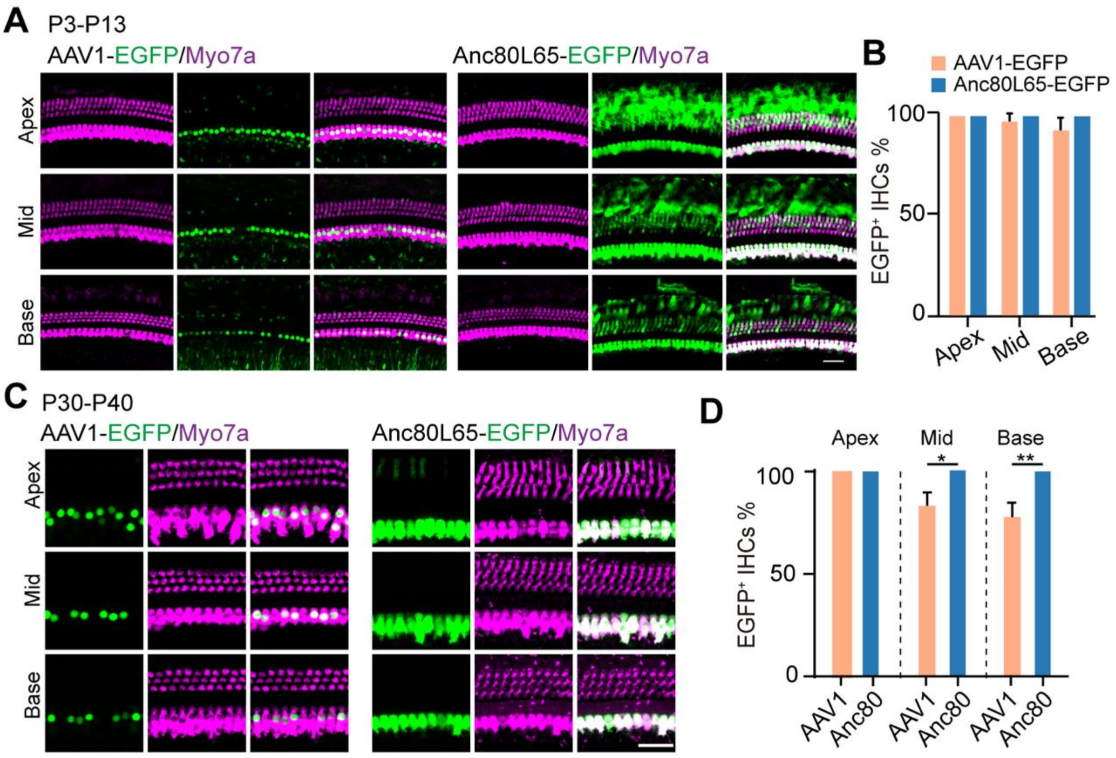

Figure S2. ***In vivo* transduction of Anc80 and AAV1 in neonatal and adult mice**. (**A**) Representative transduction in cochlea of C57 mice 10 days after delivery of Anc80 and AAV1 at P3 as indicated by the *EGFP* transgene (green). Scale bar, 50 µm. (**B**) Percentage of EGFP-positive IHCs in (**A**). (**C**) Representative confocal images of cochleae of C57 mice 10 days after delivery of Anc80 and AAV1 at P30 as indicated by the *EGFP* transgene (green). Scale bar, 30 µm. (**D**) Percentage of EGFP-positive IHCs in (**C**). Error bars indicated the standard deviation. The *p*-value was calculated by Student's *t*-test. *$p < 0.05$, **$p < 0.01$, n.s. means no significance difference.

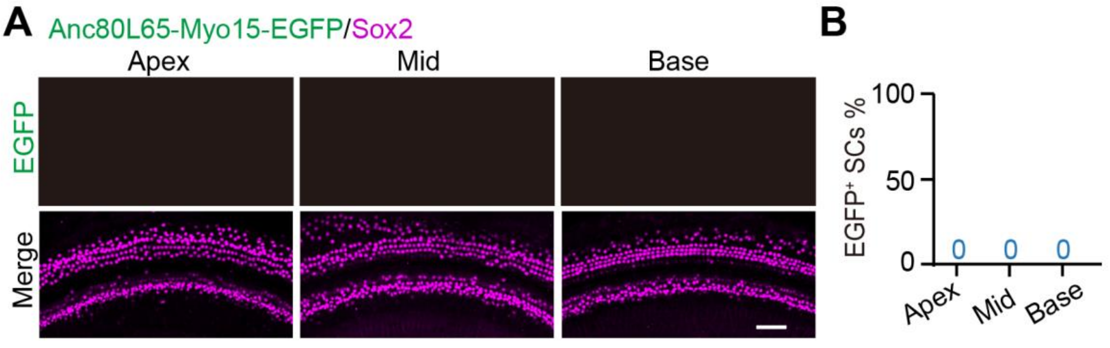

Figure S3. **The** m**Myo15 promoter does not drive EGFP expression in Sox2-positive supporting cells.** (**A**) Confocal images of cochleae of mice 10 days after delivery of Anc80-mMyo15-*EGFP* at P3. Scale bar, 50 µm. (**B**) Percentage of EGFP-positive SCs.

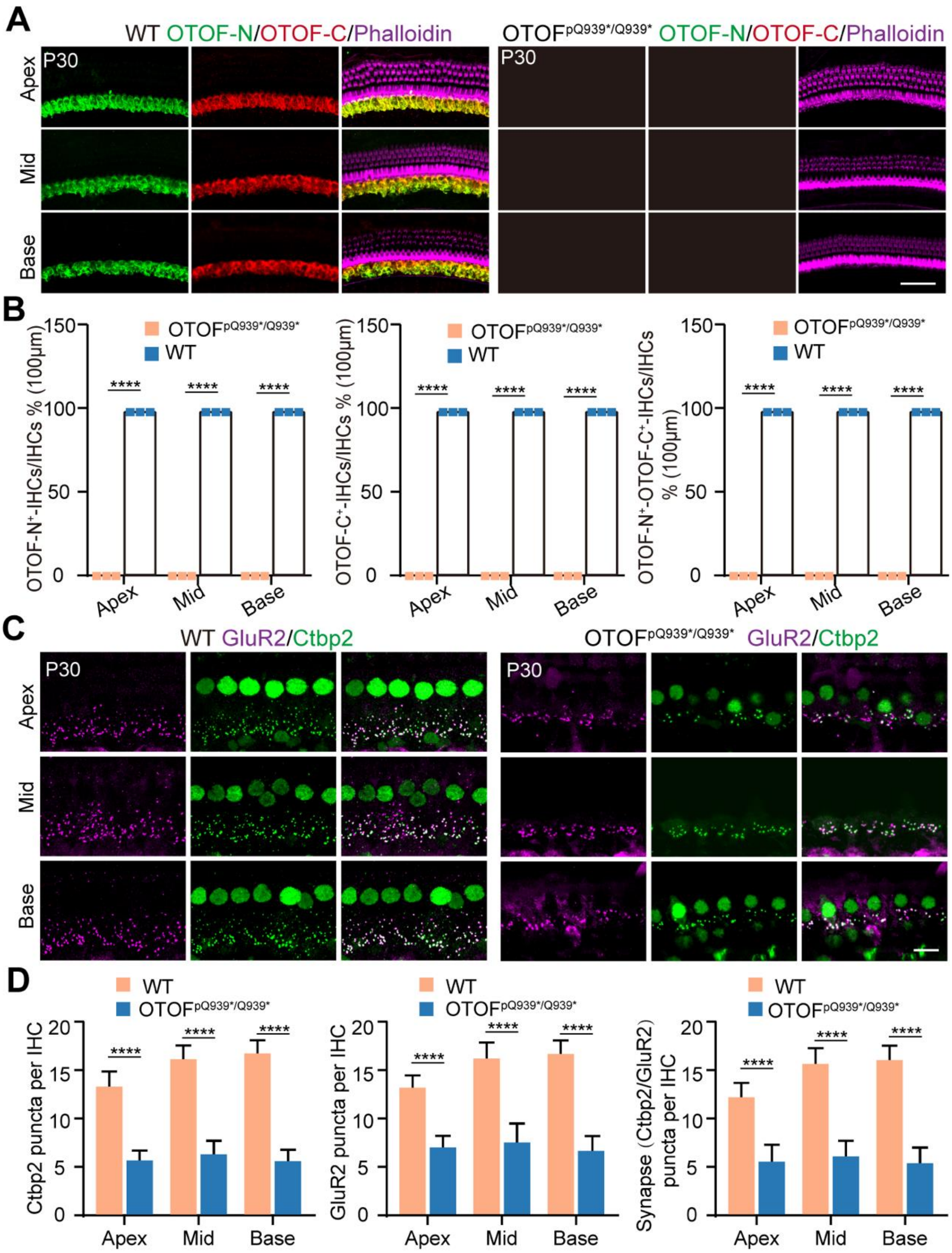

Figure S4. Characterization of OTOF$^{p.Q939*/Q939*}$ mice. (**A**) Representative confocal images from P30 cochlear sections of WT and OTOF$^{p.Q939*/Q939*}$ mice immunostained with OTOF-N (green), OTOF-C (red), and actin (magenta). Scale bar, 50 μm. (**B**) The percentages of OTOF-N, OTOF-C, and double-positive IHCs. (**C**) Representative images of synapse ribbons analyzed by immunostaining. Magenta indicates the synaptic ribbons, and green indicates the GluR2 subunit of post-synaptic glutamate receptors. Scale bar: 1 μm. (**D**) Quantification of the number of synaptic ribbons in

IHCs. Error bars indicated the standard errors of the mean. The p-value was calculated by Student's t-test. $****p < 0.0001$.

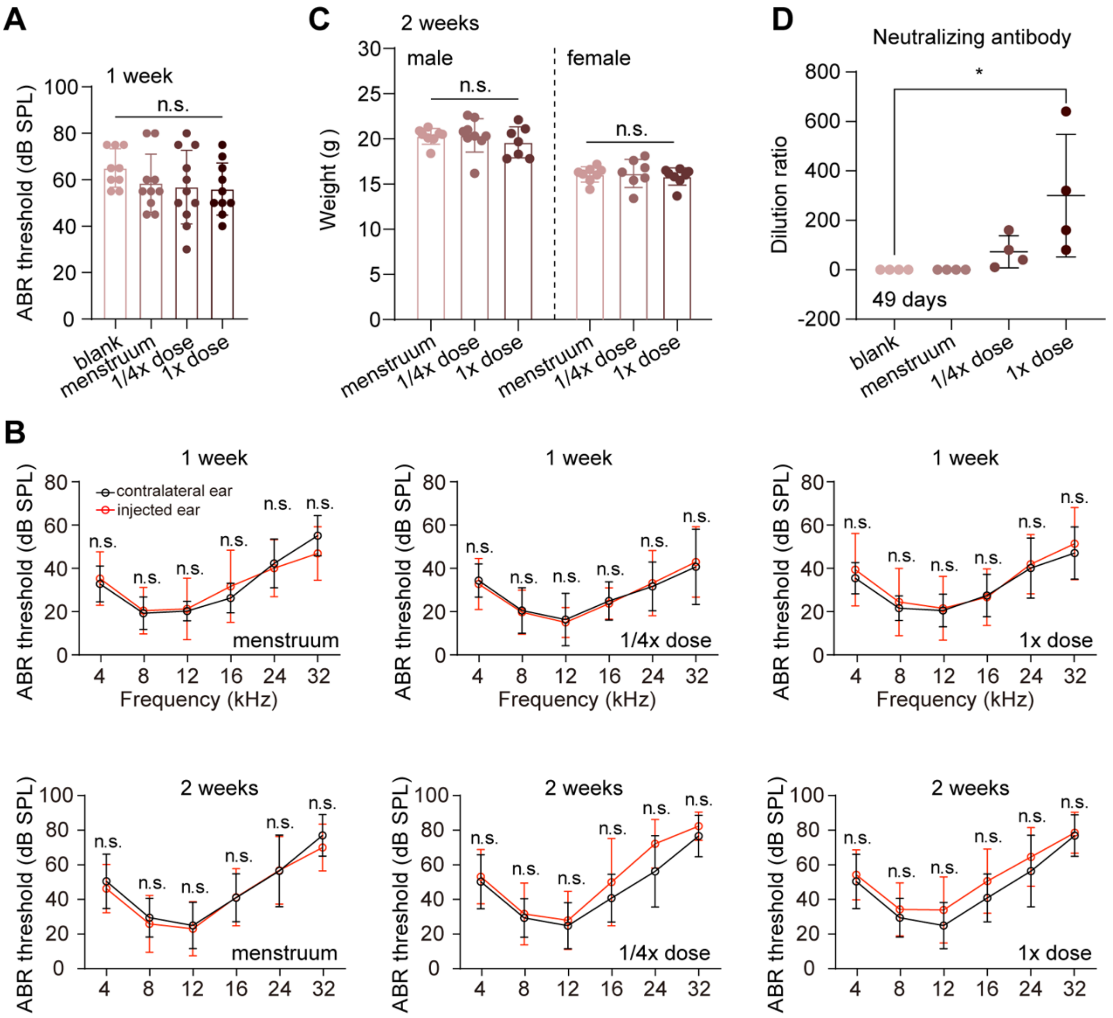

Figure S5. Safety assessment of dual-Anc80L65-*OTOF* in wild-type mice for a short-term. (**A**) Click ABR of adult wild-type mice and wild-type mice injected with menstruum, 1/4 dose, and 1× dose of AAV-*OTOF* 2 weeks later. (**B**) ABR of wild-type mice injected with menstruum and virus 2 weeks later. (**C**) The body weight of adult wild-type mice and wild-type mice injected with menstruum and virus. (**D**) The titers of neutralizing antibodies against AAV-*OTOF* 49 days post-surgery. Error bars indicated the standard deviation. The p-value was calculated by Student's t-test or one-way ANOVA. $*p < 0.05$, n.s. means no significance difference.

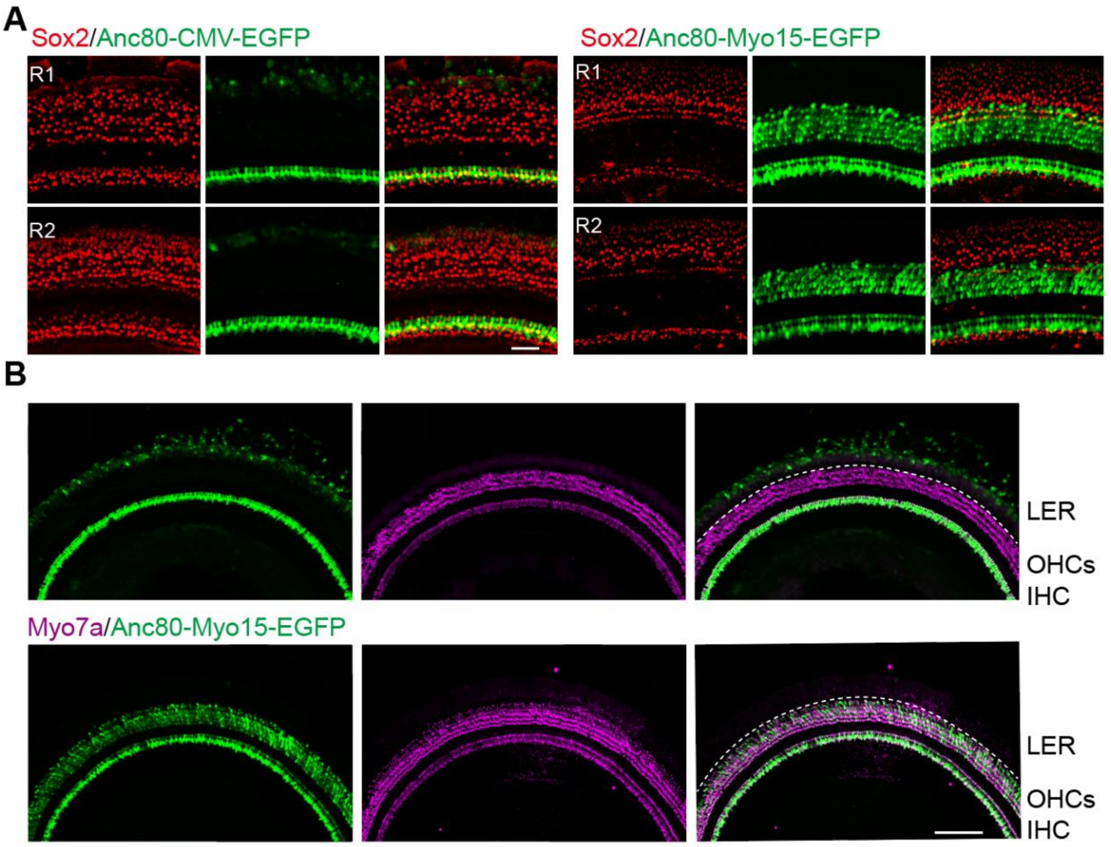

Figure S6. Both the mMyo15 and CMV promoters drive EGFP expression in the cochlear epithelia in NHPs. (**A**) Representative confocal images of cochlear supporting cells staining with Sox2 (red) and EGFP (green) transduced with Anc80L65-CMV-*EGFP* and Anc80L65-mMyo15-*EGFP*, respectively. Scale bar, 50 μm. (**B**) Representative confocal images of cochlear epithelia staining for Myo7a (magenta) and EGFP (green) after transduction with Anc80L65-CMV-*EGFP* and Anc80L65-mMyo15-*EGFP*, respectively. Cells from the lesser epithelial ridge (LER) showed no EGFP signals driven by the mMyo15 promoter. Scale bar, 100 μm.

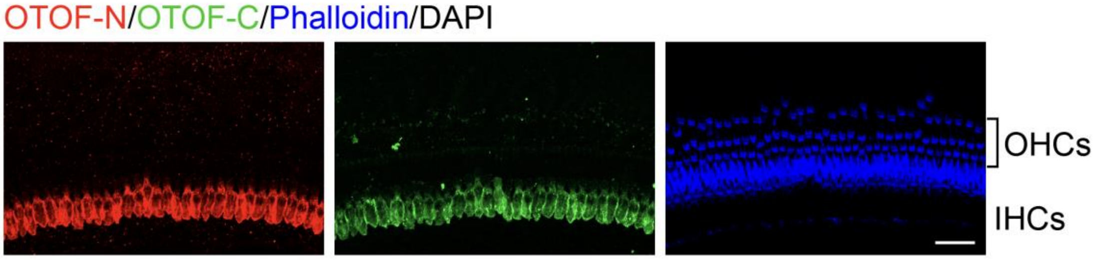

Figure S7. AAV-OTOF was not expressed in NHP OHCs driven by mMyo15 promoter. Representative confocal images of hair cells transduced with Anc80L65-mMyo15-*OTOF*-N and Anc80L65-*OTOF*-C. Z-stack images were captured and projected. Scale bar, 40 μm.

Table S1. The biodistribution of OTOF-N and OTOF-C in mouse tissues （9 days post AAV injection）

Sex	Group	Animal ID/tissue	The number of OTOF-N（copies/μg）										
			Whole blood	Left brain	Right brain	Left cochlea	Right cochlea	Brainstem	Cervical lymph nodes	Heart	Kidney	Liver	Lung
male	1/4 x dose	1-1-1	3.48E+02	5.02E+02	5.73E+01	6.62E+04	BQL	5.90E+01	1.27E+03	BQL	BQL	1.82E+04	5.20E+01
		1-1-2	4.48E+02	1.24E+03	2.39E+02	1.75E+04	1.77E+02	3.32E+03	2.27E+03	BQL	5.33E+01	3.63E+04	1.17E+02
	1 x dose	2-1-1	1.98E+02	3.16E+02	BQL	1.10E+04	BQL	4.88E+02	4.42E+02	BQL	4.58E+01	8.54E+03	BQL
		2-1-2	7.47E+02	4.64E+02	3.75E+01	4.34E+03	BQL	6.69E+01	6.51E+02	BQL	BQL	9.02E+03	BQL
female	1/4 x dose	1-2-1	BQL	BQL	BQL	1.31E+04	4.70E+01	9.66E+01	1.59E+03	BQL	BQL	1.71E+03	BQL
		1-2-2	1.24E+03	4.05E+02	4.26E+02	7.22E+04	1.14E+03	1.45E+03	1.02E+03	2.18E+02	1.31E+02	8.26E+04	7.97E+01
	1 x dose	2-2-1	2.78E+03	1.10E+03	2.16E+02	5.23E+04	2.55E+03	1.06E+04	1.34E+04	1.10E+03	3.14E+02	4.80E+05	1.56E+02
		2-2-2	3.57E+03	6.13E+03	4.80E+03	5.88E+04	2.37E+04	5.43E+04	8.97E+03	9.37E+02	6.52E+02	3.31E+05	3.69E+02

Sex	Group	Animal ID/tissue	The number of OTOF-C（copies/μg）										
			Whole blood	Left brain	Right brain	Left cochlea	Right cochlea	Brainstem	Cervical lymph nodes	Heart	Kidney	Liver	Lung
male	1/4 x dose	1-1-1	6.05E+02	6.10E+02	7.18E+01	6.38E+04	BQL	7.01E+01	9.26E+02	9.12E+01	5.09E+01	1.59E+04	7.00E+01
		1-1-2	5.84E+02	9.54E+02	1.89E+02	1.52E+04	1.51E+02	2.98E+03	7.88E+02	BQL	6.19E+01	2.60E+04	8.72E+01
	1 x dose	2-1-1	2.91E+02	4.05E+02	BQL	1.21E+04	BQL	6.69E+02	2.48E+02	BQL	7.49E+01	7.82E+03	BQL
		2-1-2	1.33E+03	5.60E+02	4.99E+01	4.11E+03	BQL	9.20E+01	5.83E+02	6.73E+01	3.41E+01	9.79E+03	4.07E+01
female	1/4 x dose	1-2-1	BQL	BQL	BQL	1.65E+04	5.28E+01	1.33E+02	2.21E+03	BQL	BQL	2.13E+03	BQL
		1-2-2	2.39E+03	4.95E+02	4.76E+02	6.18E+04	8.91E+02	1.74E+03	7.65E+02	3.49E+02	1.66E+02	5.85E+04	1.29E+02
	1 x dose	2-2-1	4.98E+03	1.32E+03	1.96E+02	5.20E+04	2.68E+03	1.26E+04	8.55E+03	1.61E+03	3.82E+02	3.97E+05	1.88E+02
		2-2-2	5.97E+03	7.84E+03	4.94E+03	7.20E+04	2.47E+04	5.31E+04	5.82E+03	1.31E+03	8.54E+02	2.96E+05	4.30E+02

Note: BQL indicates that it is lower than the lower limit of quantification. Tissue samples are measured in copies/μg, representing the number of copies of the target gene contained in the DNA sample per μg after extraction.

Table S2. Blood biochemistry in mice（9 days post AAV injection）

	Blank	Menstruum	1/4 x dose	1 x dose
Alanine aminotransferase (U/L)	24	24	24	28
	32	24	34	26
	22	28	24	22
	22	22	38	28
Aspartate aminotran sferase (U/L)	46	52	50	52
	94	46	64	48
	64	56	50	60
	50	58	86	68
Total protein (g/L)	55.2	53.4	55	51.8
	56.6	53.4	54.4	54
	53.8	55.8	51.6	52.8
	50.8	56.6	56.2	51.2
Albumin (g/L)	26.4	25.8	24.2	24.2
	25.4	25.2	23	25.6
	26.8	27.2	25.8	26.6
	26	27.6	23.2	26
Alkaline phosphatase (U/L)	74	80	70	84
	78	82	128	110
	106	124	122	110
	98	116	176	108
Glucose (mm/L)	15.42	11.38	12.48	8.8
	12.92	10.52	12.16	13.54
	7.78	11.76	11.42	6.2
	14.44	8.86	7.04	6.76
Urea (mm/L)	7.6	9.4	8.4	10
	8.8	10.6	7.2	9.2
	8.2	8.2	9.4	8.4
	7.6	9.6	10.4	10.2
Creatine kinase (U/L)	86	302	90	134
	224	100	178	80
	144	124	216	104
	116	262	252	156
Total bilirubin(μm/L)	1.68	2.3	1.96	2.04
	2.46	1.94	1.86	1.76
	1.54	2.12	2.8	1.36
	1.26	1.8	1.18	1.22
Gamma-glutamyltranspept idase (U/L)	0	0	0	0
	0	0	0	0
	0	0	0	0
	0	0	0	0
Lactic dehydrogenase (U/L)	276	330	408	344
	374	308	404	256
	316	362	264	264
	306	316	464	268
Total cholesterol	2.84	2.44	2.3	2.76
	3.06	2.48	2.58	2.7

(mm/L)	2.18	2.12	2	2.14
	2.16	2.08	2.22	2.18
Triglyceride (mm/L)	0.56	0.58	0.6	0.7
	0.7	0.8	0.7	0.52
	0.6	0.62	0.72	0.58
	0.62	0.52	0.72	0.54
Calcium (mm/L)	3.38	3.66	3.78	3.48
	3.46	3.34	3.34	3.42
	3.44	3.28	3.16	3.66
	3.4	3.58	2.96	3.48
Phosphorus (mm/L)	2.34	2.26	1.94	2.34
	2.32	2.38	2.08	2.16
	2.52	2.4	2.56	2.38
	2.74	2.34	2.28	2.3
Albumin/globulin	0.9	0.9	0.8	0.9
	0.8	0.9	0.7	0.9
	1	1	1	1
	1	1	0.7	1
Creatinine(μm/L)	12	12	8	12
	12	10	12	8
	14	10	12	10
	16	14	12	10
Sodium concentration (mm/L)	140.8	144.2	144.4	143
	144.8	145.4	143.6	145.2
	145.8	143.8	141.8	145.6
	143.6	142.4	142.8	144.6
Potassium ion concentration (mm/L)	4.66	5.62	5.56	5.5
	5.44	5.68	5.5	5.1
	5.08	5.32	5.02	5.52
	4.84	5.84	5.44	5.1
Chloride ion concentration (mm/L)	104.2	107.8	107.2	108.6
	107.6	109.2	106.4	109
	112	109	108.4	111.8
	109	109.6	110.6	111.6

Table S3. Blood cell biochemistry in mice（9 days post AAV injection）

	Blank	Menstruum	1/4 x dose	1 x dose
White blood cells (×9/L)	4.31	3.07	2.78	5.97
	3.9	4.58	4.15	3.85
	2.1	3.47	4.78	2.59
	2.77	3.65	3.34	5.04
Red blood cells (×12/L)	10.72	10.96	10.14	10.48
	8.87	10.69	10.48	10.76
	10.72	10.2	9.99	10.39
	10.65	10.65	11.13	10.71
Hemoglobin (×g/L)	152	156	144	144
	128	153	145	149
	151	145	130	150
	153	149	161	153
Red blood cell specific volume (%)	48.7	49.2	45	44.9
	40	47.5	46.1	47.2
	47.4	45.6	42.1	46.1
	47.2	47	49.9	47.6
Mean corpuscular volume (×pg)	45.4	44.9	44.4	42.8
	45.1	44.4	44	43.9
	44.2	44.7	42.1	44.4
	44.3	44.1	44.8	44.4
Mean erythrocyte hemoglobin (×pg)	14.2	14.2	14.2	13.7
	14.4	14.3	13.8	13.8
	14.1	14.2	13	14.4
	14.4	14	14.5	14.3
Mean erythrocyte hemoglobin concentration (×g/L)	312	317	320	321
	320	322	315	316
	319	318	309	325
	324	317	323	321
Platelet count (×9/L)	1331	1250	1290	1551
	1111	1177	1344	1378
	1235	1164	1619	1140
	1285	1300	1321	1325
Lymphocyte (%)	87.9	82.4	83.5	72.2
	85.4	82.1	85.1	83.4
	84.3	72	72.6	87.6
	82.3	83.6	80.5	76.6
Monocyte (%)	2.1	2.3	1.8	2.2
	2.1	3.9	1.7	1.3
	1.9	2.6	4.4	2.3
	1.8	2.5	2.1	1.8
Neutrophils (%)	9.1	13.3	12.9	24.6
	12	10.5	12	15
	13.3	24.5	22	9.7
	14.5	12.5	14.4	21
Eosinophils (%)	0.7	2	1.8	1
	0.5	3.5	1.2	0.3

Eosinophils (%)	0.5	0.6	0.8	0.4
	1.4	1.4	3	0.6
Basophils (%)	0.2	0	0	0
	0	0	0	0
	0	0.3	0.2	0
	0	0	0	0
Lymphocyte (absolute value) (×9/L)	3.79	2.53	2.32	4.31
	3.33	3.76	3.53	3.21
	1.77	2.5	3.47	2.27
	2.28	3.05	2.69	3.86
Monocyte (absolute value) (×9/L)	0.09	0.07	0.05	0.13
	0.08	0.18	0.07	0.05
	0.04	0.09	0.21	0.06
	0.05	0.09	0.07	0.09
Neutrophils (absolute value) (×9/L)	0.39	0.41	0.36	1.47
	0.47	0.48	0.5	0.58
	0.28	0.85	1.05	0.25
	0.4	0.46	0.48	1.06
Eosinophils (absolute value) (×9/L)	0.03	0.06	0.05	0.06
	0.02	0.16	0.05	0.01
	0.01	0.02	0.04	0.01
	0.04	0.05	0.1	0.03
Basophils (absolute value) (×9/L)	0.01	0	0	0
	0	0	0	0
	0	0.01	0.01	0
	0	0	0	0
RDW-CV (%)	19.4	18.6	19	19.8
	17.6	18.9	19.1	19.1
	19.4	19.9	21	18.2
	19.7	19.4	19.2	19.2
RDW-SD (%)	24.7	22.2	23.8	24.1
	24.1	23	23.1	22.1
	22.9	25.7	26.9	22.1
	24	23.5	22.7	23.8
Platelet distribution width (fL)	6.3	6.1	6.1	6.2
	6	6	6.2	6.2
	6.2	6.2	6.2	6.4
	6.2	6.3	6.4	6.2
Mean platelet volume (fL)	5.9	5.9	5.9	6.1
	6.1	5.8	6.1	6
	6	6.1	6.3	6.1
	5.9	5.9	6	6.1
Reticulocyte count (%)	4.42	3.7	3.78	4.51
	4.57	3.83	3.77	4.64
	4.33	3.18	5.38	3.03
	4.16	3.58	3.66	3.97
Reticulocyte (absolute value) (×12/L)	0.4738	0.4055	0.3833	0.4726
	0.4054	0.4094	0.3951	0.4993
	0.4642	0.3244	0.5375	0.3148

	0.443	0.3813	0.4074	0.4252
Platelet accumulation (%)	0.79	0.74	0.77	0.94
	0.68	0.68	0.81	0.83
	0.74	0.71	1.02	0.7
	0.76	0.77	0.79	0.81
Nucleated red cells (%)	0.5	0.3	0.4	0.2
	0.5	0.2	0.5	0
	0.5	0.3	0.4	0
	0.4	0.3	0.3	0.2
Nucleated erythrocyte (absolute value) (×9/L)	0.02	0.01	0.01	0.01
	0.02	0.01	0.02	0
	0.01	0.01	0.02	0
	0.01	0.01	0.01	0.01

Table S4. Blood cell biochemistry in cynomolgus monkey

		Control group		Experimental group	
Animal ID		1	2	3	4
White blood cells	$\times10^3$/uL	11.77	10.47	13.68	20.38
Neutrophils	%	42.8	57.8	49.3	59.4
	$\times10^3$/uL	5.03	6.05	6.74	12.11
Lymphocyte	%	51.8	38.2	45.8	37.4
	$\times10^3$/uL	6.09	4	6.27	7.61
Monocyte	%	3.1	3	3.1	2.4
	$\times10^3$/uL	0.36	0.32	0.42	0.5
Eosinophil	%	1.2	0.5	0.6	0.1
	$\times10^3$/uL	0.15	0.05	0.08	0.02
Basophil	%	0.5	0.3	0.3	0.4
	$\times10^3$/uL	0.06	0.03	0.05	0.07
Large unstained cell	%	0.6	0.2	0.9	0.3
	$\times10^3$/uL	0.08	0.03	0.12	0.06
Red blood cells	×106/uL	5.91	5.25	5.58	5.57
Hemoglobin	g/dL	14.6	12.4	13.3	14.3
Red blood cell specific	%	45.8	40.4	41.8	46.5
Mean corpuscular volume	fL	77.5	77	74.8	83.5
Mean corpuscular hemoglobin	Pg	24.7	23.5	23.9	25.7
Mean corpuscular hemoglobin	g/dL	31.9	30.6	31.9	30.8
Platelet	$\times10^3$/uL	357	420	277	417
Red blood cell distribution	%	16.3	18.2	17.3	17.1

Table S5. Blood biochemistry in cynomolgus monkey

	Control group		Experimental group	
Animal ID	1	2	3	4
Glutamic-pyruvic transaminase (U/L)	49.4	56	36.5	41.1
Glutamic oxalacetic transaminase(U/L)	55.8	60	60.1	60.6
Total protein (g/L)	73.2	71.4	69.6	81.9
Albumin (g/L)	43.7	45.3	43.8	49.1
Alkaline phosphatase (U/L)	465.7	507.4	441.5	494.3
Total bilirubin (μmol/L)	3.03	1.95	2.61	2.68
Serum total cholesterol (μmol/L)	3.31	4.77	3.5	2.82
Triglyceride (μmol/L)	0.46	0.25	0.21	0.37
Urea (μmol/L)	9.75	8.05	6.63	7.16
Crea (μmol/L)	85.3	74.9	59.8	77
Creatine kinase (μmol/L)	289.3	259.3	238.7	422.7
Lactate dehydrogenase (U/L)	386	359	309	303
C-reactive protein (mg/mL)	1.912	1.583	1.718	3.611
